# Nitro-fatty acids suppress ischemic ventricular arrhythmias by preserving calcium homeostasis

**DOI:** 10.1038/s41598-020-71870-6

**Published:** 2020-09-18

**Authors:** Martin Mollenhauer, Dennis Mehrkens, Anna Klinke, Max Lange, Lisa Remane, Kai Friedrichs, Simon Braumann, Simon Geißen, Sakine Simsekyilmaz, Felix S. Nettersheim, Samuel Lee, Gabriel Peinkofer, Anne C. Geisler, Bianca Geis, Alexander P. Schwoerer, Lucie Carrier, Bruce A. Freeman, Matthias Dewenter, Xiaojing Luo, Ali El-Armouche, Michael Wagner, Matti Adam, Stephan Baldus, Volker Rudolph

**Affiliations:** 1 Clinic III for Internal Medicine, Department of Cardiology, Center for Molecular Medicine Cologne (CMMC), University of Cologne, Kerpener Str. 62, 50937 Cologne, Germany; 2 Center for Molecular Medicine Cologne, CMMC, University of Cologne, Cologne, Germany; 3 Clinic for General and Interventional Cardiology/ Angiology, Herz- Und Diabeteszentrum NRW, Ruhr-Universitaet Bochum, Bad Oeynhausen, Germany; 4 General and Interventional Cardiology University Heart Center Hamburg, University Hospital Hamburg-Eppendorf (UKE), Hamburg, Germany; 5 Department of Cellular and Integrative Physiology, University Medical Center Hamburg Eppendorf, DZHK (German Centre of Cardiovascular Research), Partner Site Hamburg/Kiel/Lübeck, Hamburg, Germany; 6 Experimental Pharmacology and Toxicology, University Hospital Hamburg-Eppendorf (UKE), Hamburg, Germany; 7 Department of Pharmacology and Chemical Biology, University of Pittsburgh, Pittsburgh, PA USA; 8 Institute of Experimental Cardiology, University of Heidelberg, Heidelberg, Germany; 9 German Centre for Cardiovascular Research (DZHK), Partner Site, Heidelberg/Mannheim, Germany; 10 Department of Pharmacology and Toxicology, Technische Universitaet Dresden, Dresden, Germany; 11 Clinic for Internal Medicine and Cardiology, Heart Center Dresden, Dresden, Germany

**Keywords:** Ventricular tachycardia, Calcium signalling

## Abstract

Nitro-fatty acids are electrophilic anti-inflammatory mediators which are generated during myocardial ischemic injury. Whether these species exert anti-arrhythmic effects in the acute phase of myocardial ischemia has not been investigated so far. Herein, we demonstrate that pretreatment of mice with 9- and 10-nitro-octadec-9-enoic acid (nitro-oleic acid, NO_2_-OA) significantly reduced the susceptibility to develop acute ventricular tachycardia (VT). Accordingly, epicardial mapping revealed a markedly enhanced homogeneity in ventricular conduction. NO_2_-OA treatment of isolated cardiomyocytes lowered the number of spontaneous contractions upon adrenergic isoproterenol stimulation and nearly abolished ryanodine receptor type 2 (RyR2)-dependent sarcoplasmic Ca^2+^ leak. NO_2_-OA also significantly reduced RyR2-phosphorylation by inhibition of increased CaMKII activity. Thus, NO_2_-OA might be a novel pharmacological option for the prevention of VT development.

## Introduction

Due to significant advances in the therapy of acute myocardial ischemia (AMI), e.g. more aggressive approaches to coronary revascularization and secondary prevention, AMI mortality has declined drastically over the last decades^1^. However, patients suffering from AMI with ventricular tachycardia (VT) or -fibrillation (VF), still have a fourfold increased risk of in-hospital mortality^2^.

The mechanisms accounting for the development of acute VT are diverse: in the acute ischemic phase, 15–30 min after AMI, the development of so called 1B arrhythmias has been linked to increased catecholamine release and disturbances in Ca^2+^ signaling within cardiomyocytes of the ischemic border zone, thus leading to ventricular conduction blocks and enhanced VT vulnerability^3,4^. In particular, oxidation and subsequent autophosphorylation of Ca^2+^/calmodulin dependent kinase II (CaMKII) cause malfunctioning of ryanodine receptor type 2 (RyR2) channels and dysregulation of phospholamban (PLN)-dependent cytosolic Ca^2+^ clearance leading to an intracellular Ca^2+^ leak from the sarcoplasmic reticulum (SR)^5,6^. Consequently, depolarization of the membrane potential is enhanced thereby causing inactivation of Na^+^ channels with further slowing of ventricular conduction^7^.

As the identification of AMI patients at risk for VT remains difficult, a therapeutic strategy would have to be applied to a broad patient population. Therefore, dietary intervention with endogenously produced anti-arrhythmic modulators appears as an attractive therapeutic approach. This concept has been recently supported by the randomized double blind trial *REDUCE-IT* which demonstrates that prophylactic treatment with icosapentaenoic acid-ethyl (VASCEPA) significantly reduces cardiovascular events, the rate of cardiac arrests, and the number of sudden cardiac deaths (SCD)^8^.

Nitro-oleic acid (9- and 10-nitro-octadec-9-cis-enoic acid; NO_2_-OA) is an endogenously generated unsaturated fatty acid nitroalkene derivative that is formed by oleic acid reaction with nitric oxide and nitrite-derived nitrogen dioxide (•NO_2_)^9,10^. NO_2_-OA is conferred with an electrophilic β-carbon that rapidly and reversibly reacts with nucleophilic cysteine-, and to a lesser extent histidine, residues via a Michael addition^11^. The covalent nitro adduct is essential for the metabolic effects of NO_2_-OA since native oleic acid is not electrophilic and ineffective in various studies^12–14^. Nitro-fatty acids have been identified as potent endogenous anti-inflammatory mediators that affect a number of signaling mediators, transcriptional regulatory proteins and ion channels, including nuclear factor ‘kappa-light-chain-enhancer’ of activated B-cells (NF-κB), kelch-like ECH-associated protein 1 (Keap1/Nrf2), mitogen-activated protein kinases (MAPK), peroxisome proliferator-activated receptor gamma (PPARγ) and transient receptor potential channels (TRP) channels^15–17^. Preclinical studies have demonstrated therapeutic benefit in a variety of disease models such as pulmonary hypertension, nephritis and, of relevance to herein, reperfusion damage after myocardial ischemia (I/R) and a subsequent attenuation of cardiac fibrosis, reduced infarct size and preserved left ventricular function^18–20^. Since NO_2_-OA has passed safety clinical phase I trials^21^ these results have been translated into two, currently recruiting, clinical phase II trials targeting pulmonary hypertension and primary focal segmental glomerular sclerosis^22,23^.

Very low levels of NO_2_-OA could be detected in human serum- and urine^24^ as well as in the myocardium of rodents^19^. Nonetheless, inflammatory stimuli like I/R induce substantial generation of NO_2_-OA up to µM concentrations by enhanced formation of nitrating species^19^. Furthermore, increased endogenous formation of nitro-linoleic acid has been demonstrated in healthy humans after dietary supplementation with conjugated linoleic acid and nitrite or nitrate^25^.

If anti-arrhythmic effects of nitro-fatty acids could indeed be shown, a dietary supplementation or a pure pharmaceutical-based nitro-fatty acid therapeutic strategy in patients at risk for cardiovascular events could evolve as a strategy for prevention of sudden cardiac death.

## Results

### Susceptibility to VT upon right ventricular stimulation

**Figure 1:**
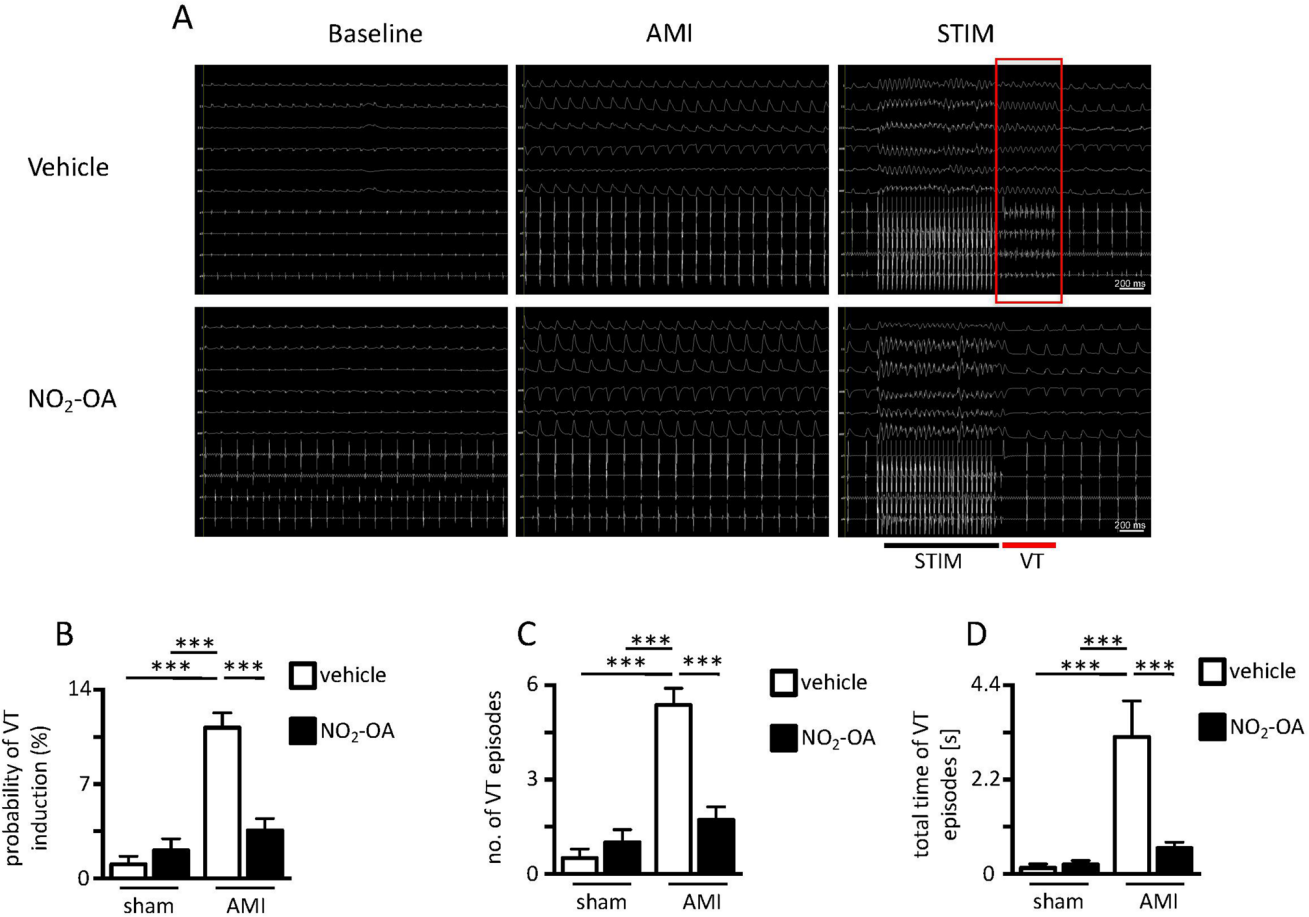
Surface and intracardiac electrocardiograms (ECG) of NO_2_-OA versus vehicle treated mice. (**A**) Representative images of ECG before induction of myocardial ischemia (Baseline; left panel), 20 min after ischemia induction without right ventricular stimulation (AMI; middle panel) and after right ventricular stimulation (STIM; right panel) with appearance of ventricular tachycardias (VT) in NO_2_-OA- or vehicle treated mice. Scale bar = 200 ms. (**B**) Probability of VT in sham versus LAD-ligated mice (AMI) with and without NO_2_-OA treatment (AMI vehicle: 11.2 ± 3.1% vs. AMI NO_2_-OA: 3.6 ± 2.3; n = 8/7). (**C**) Number of VT episodes (AMI vehicle: 5.4 ± 1.5 vs. AMI NO_2_-OA: 1.7 ± 1.1; n = 8/7) and (**D**) Total time of VT episodes in sham versus AMI mice with and without NO_2_-OA treatment (AMI vehicle: 3.2 ± 2.4 s vs. AMI NO_2_-OA: 0.6 ± 0.4 s; n = 8/7). Graphs show Mean ± SEM. Brackets indicate Mean ± SD. *** P < *0.001.*

Susceptibility to VT induction 20 min after ligation of the left anterior descending artery (LAD) was drastically reduced in animals pretreated with NO_2_-OA (representative ECG recordings shown in Fig. 1A). Thus, the probability of VT induction, calculated as the percentage of induced VT episodes per stimulation maneuvers (Fig. 1B), as well as the total number of inducible VT episodes (Fig. 1C) were both significantly lower in NO_2_-OA pretreated animals. Additionally, the total time of VT episodes was reduced after NO_2_-OA-pretreament (Fig. 1D) indicating that VT episodes in NO_2_-OA pretreated ischemic hearts were rapidly self-terminating.

### Homogeneity of conduction

**Figure 2:**
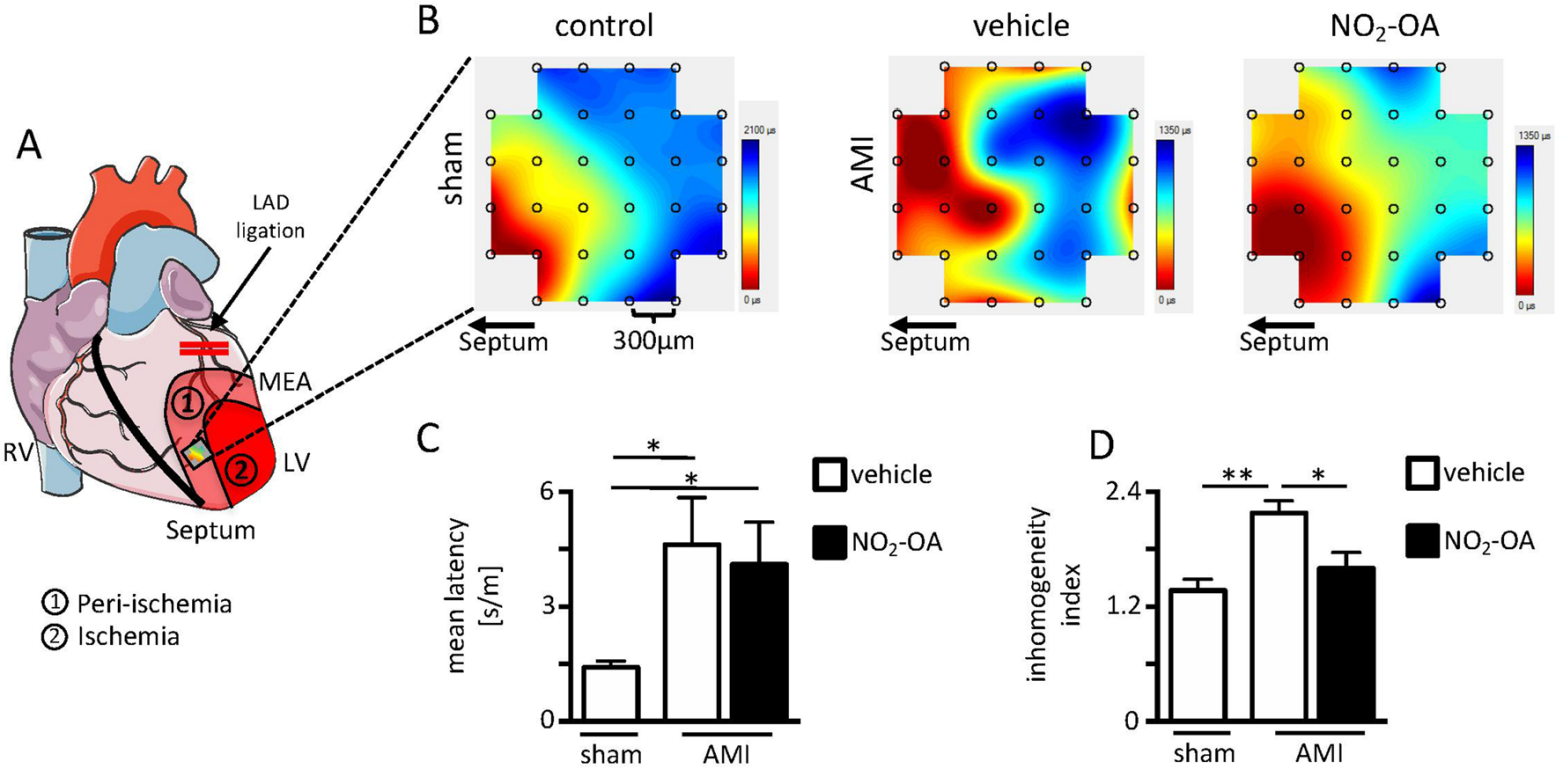
Epicardial mapping analyses within the peri-ischemic region by microelectrode array. (**A**) Scheme of left ventricular positioning of the multi-electrode array (MEA) indicating ischemic and peri-ischemic regions and cardiac electrical conduction (blue arrows). (**B**) Representative maps of spontaneous conduction in the peri-ischemic region with vehicle treatment (control) versus treatment with NO_2_-OA. Left side of the maps indicate electrodes oriented nearest to myocardial septum as shown in A. (**C**) Mean inter-electrode latency of electrical conduction (sham vehicle: 1.4 ± 0.5 s/m vs. AMI vehicle: 4.6 ± 3.3 s/m vs. AMI NO_2_-OA: 4.1 ± 2.9 s/m; n = 8/7/7) and (**D**) inhomogeneity index in sham animals versus 20 min of ischemia with and without NO_2_-OA treatment (sham vehicle: 1.4 ± 0.3 vs. AMI vehicle: 2.2 ± 0.3 vs. AMI NO_2_-OA: 1.6 ± 0.4; n = 8/7/7). Interelectrode distance = 300 µm. Graphs show Mean ± SEM. Brackets indicate Mean ± SD. * P < 0.05; ** P < *0.01.* Figures were produced using Servier Medical Art (https://www.servier.com/).

Epicardial mapping studies within the peri-ischemic region (positioning of the multi-electrode array (MEA) is shown in Fig. 2A) clearly revealed a more homogeneous conduction pattern in NO_2_-OA pretreated- compared to vehicle treated animals (representative conduction maps are shown in Fig. 2B). Of interest, only weak and therefore unevaluable electrical signals were detectable within the core ischemic region (data not shown). The mean electrical latency, measured apico-septally next to the ischemic core region^26^, was significantly elevated after ischemia induction, indicating a reduction of electrical conduction velocity. Although latency was numerically lower in NO_2_-OA pretreated hearts as compared to controls, it failed to reach statistical significance (Fig. 2C). However, the index of inhomogeneity was significantly lower upon administration of NO_2_-OA, indicating a reduction in ventricular conduction blocks (Fig. 2D)^27^.

To investigate potential side effects of NO_2_-OA on action potential (AP) development, we performed patch-clamp analysis of isolated adult cardiomyocytes under baseline conditions. The treatment with NO_2_-OA revealed no differences in the AP morphology regarding resting potential (Supplemental Fig. 1A), AP overshoot, (Supplemental Fig. 1B) or AP duration (APD, Supplemental Fig. 1C) between control and NO_2_-OA treated cells.

### Ca^2+^ transient analyses under isoproterenol treatment

**Figure 3:**
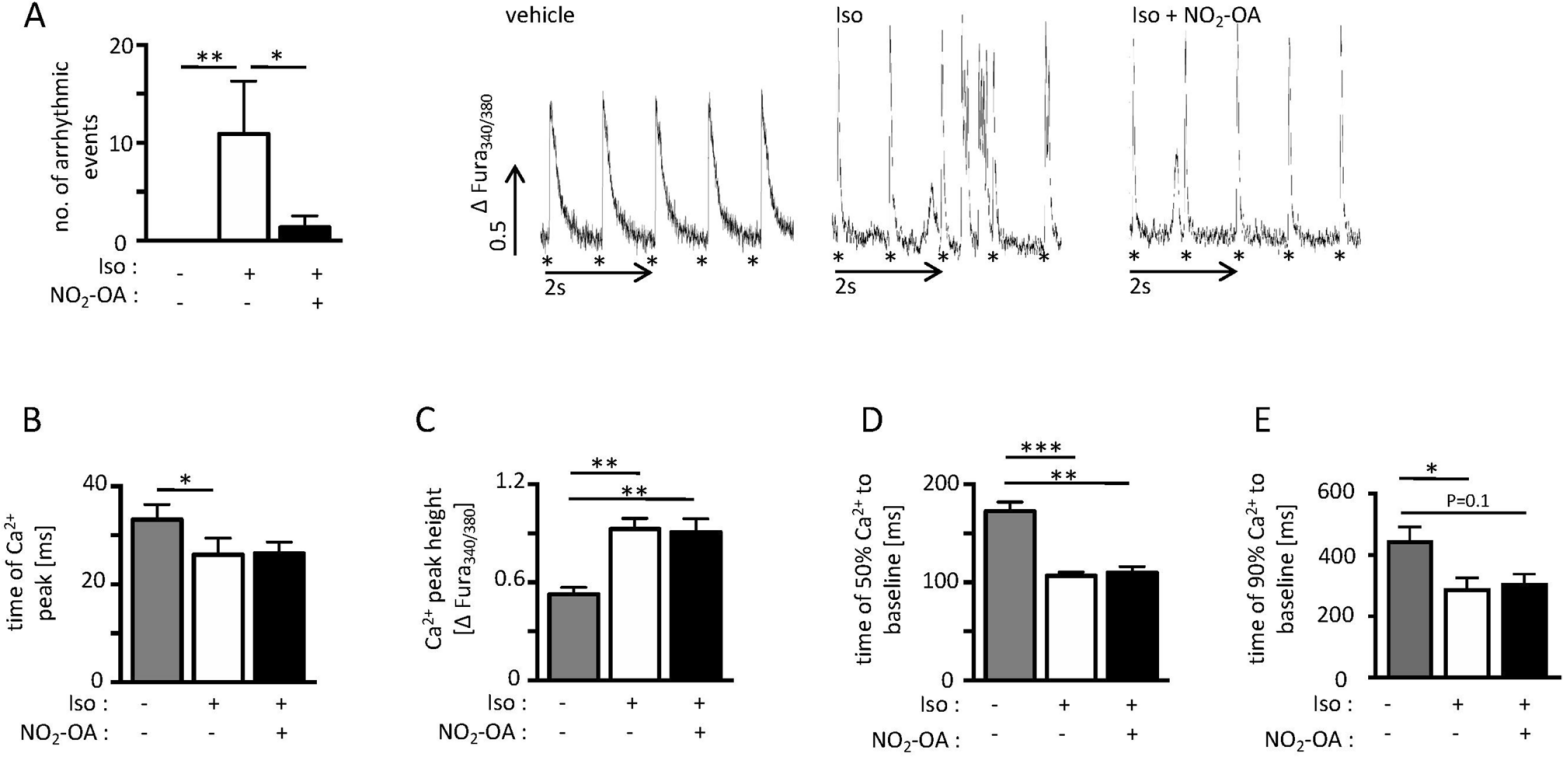
Rhythm analyses and Ca^2+^ transient measurements of isolated adult cardiomyocytes under beta-adrenergic stimulation. (**A**) Number of arrhythmic events and representative recordings of calcium transients of isolated adult cardiomyocytes with and without Iso and Iso + NO_2_-OA treatment (vehicle: 0.0 ± 0.0 vs. Iso: 10.9 ± 18.6 vs. Iso + NO_2_-OA: 1.4 ± 3.9; n(cells out of 3 animals) = 9/12/11). Asterisks indicate time point of external stimulation. (**B)** Time to maximum Ca^2+^ peak as indicator for Ca^2+^ influx velocity (vehicle: 33.2 ± 9.3 ms vs. Iso: 26.1 ± 13.1 ms vs. Iso + NO_2_-OA: 26.3 ± 8.4 ms; n(cells out of 3 animals) = 9/15/13). (**C**) Relative Ca^2+^ peak height as indicator for the maximal amount of Ca^2+^ in control- versus Iso- and Iso + NO_2_-OA treated cells (vehicle: 0.53 ± 0.12 vs. Iso: 0.93 ± 0.25 vs. Iso + NO_2_-OA: 0.91 ± 0.29 ms; n(cells out of 3 animals) = 9/15/13). (**D**) Time to 50% Ca^2+^ decay (vehicle: 173.7 ± 35.9 ms vs. Iso: 106.4 ± 22.8 ms vs. Iso + NO_2_-OA: 89.9 ± 17.8 ms; n(cells out of 3 animals) = 9/14/13) and € 90% Ca^2+^ decay as indicators for cytosolic Ca^2+^ clearance (vehicle: 448.0 ± 12.5 ms vs. Iso: 291.1 ± 12.8 ms vs. Iso + NO_2_-OA: 308.0 ± 10.3 ms; n (cells out of 3 animals) = 8/14/12). Graphs show Mean ± SEM. Brackets indicate Mean ± SD. *P < 0.05; **P < 0.01; ***P < *0.001.*

Given that catecholamine induced disturbances in Ca^2+^ homeostasis are a major contributor to arrhythmia development in acute ischemia^3^, we investigated cytosolic Ca^2+^ transients and arrhythmic events in isolated adult cardiomyocytes by ratiometric fluorescence imaging (IonOptix Corp)^28^. The number of arrhythmic events which appeared aside from a basic 1 Hz pacing stimulus (e.g. caused by early after depolarizations (EAD) and delayed after depolarizations (DAD)), was increased after beta-adrenergic stimulation with isoproterenol (Iso)^3^. Crucially, this increase was prevented by NO_2_-OA treatment (Fig. 3A). Further investigation of single cellular calcium transients revealed that the time to Ca^2+^ peak concentration upon Iso challenge (Fig. 3B) was reduced after NO_2_-OA treatment indicating a faster cytosolic Ca^2+^ influx. Furthermore, an increased peak height of the Ca^2+^ transient (Fig. 3C) was noted in both Iso treated groups as compared to vehicle treatment indicating elevated cytosolic Ca^2+^ levels. Accordingly, adrenergic stimulation led to a faster cytosolic Ca^2+^ clearance, as indicated by the reduced time to reach 50% or 90% of the maximum cellular Ca^2+^ concentration relative to baseline levels (Fig. 3D, E). Of note, no differences by additional NO_2_-OA treatment were detected.

NO_2_-OA treatment of isolated adult cardiomyocytes under baseline conditions furthermore did not influence the characteristics of Ca^2+^ transients and intracellular Ca^2+^ levels (Supplementary Figure S2).

Given the reduced appearance of spontaneous arrhythmic events upon NO_2_-OA treatment after Iso stimulation, we next investigated RyR2-dependent sarcoplasmic calcium leak as a potential pro-arrhythmic molecular mechanism.

### Ca^2+^ leak analyses under isoproterenol treatment

**Figure 4:**
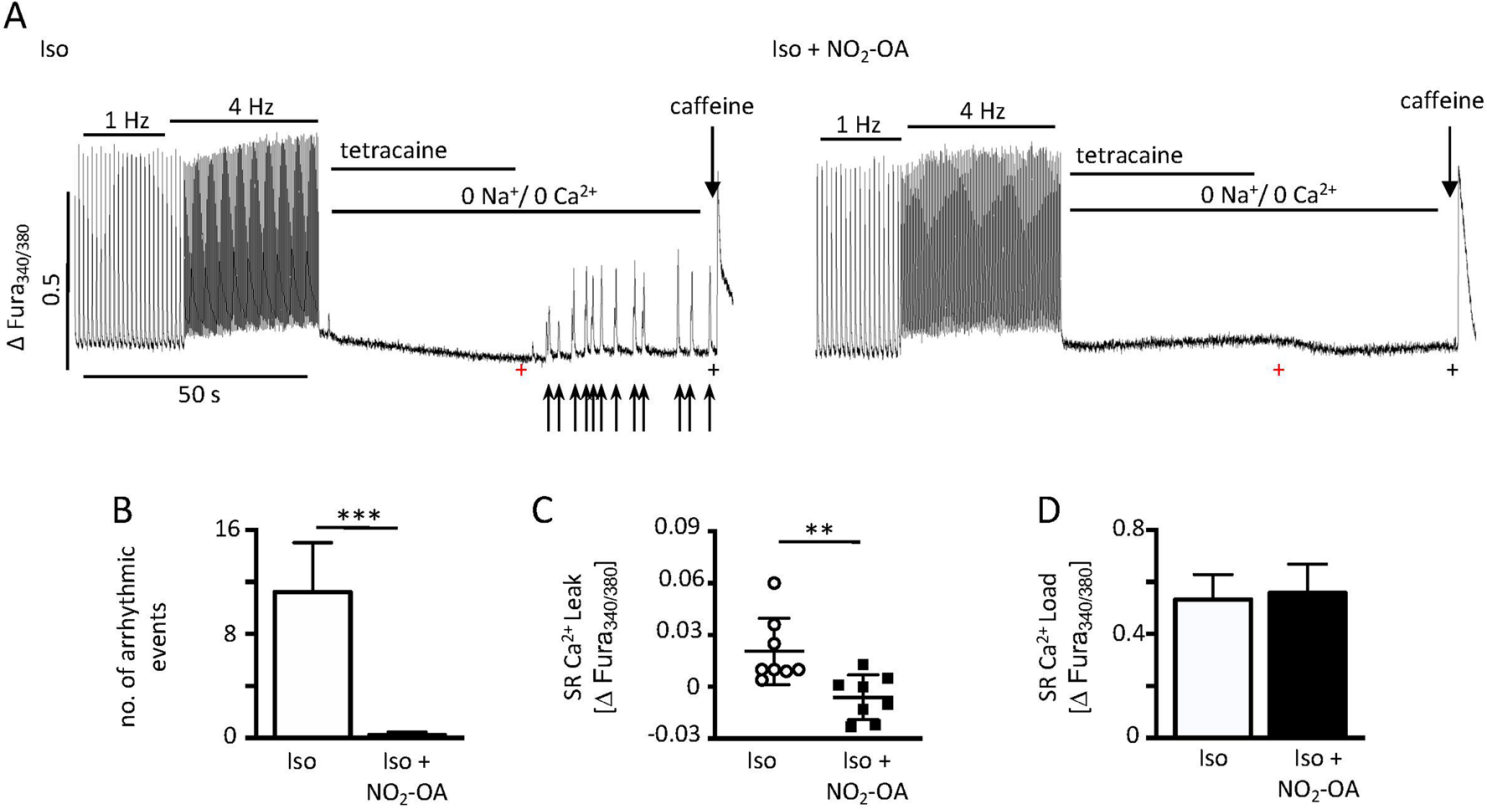
Iso dependent Ca^2+^ leak analyses in isolated adult cardiomyocytes. (**A**) Representative Ca^2+^ transient recordings of isolated adult isoproterenol (Iso)- and Iso + NO_2_-OA treated cardiomyocytes upon field stimulation (1 Hz and 4 Hz) followed by assessment of Ca^2+^ leak under non-stimulated conditions after removal of the RyR2 inhibitor tetracaine. Arrows indicate spontaneous arrhythmic events (red and black crosses visualize time points of Fura-2 ratios used for Ca^2+^ leak calculation). (**B**) Total number of arrhythmic events after removal of tetracaine (between red and black crosses) of Iso or Iso + NO_2_-OA subjected isolated cardiomyocytes (Iso: 11.2 ± 11.42 vs. Iso + NO_2_-OA: 0.2 ± 0.67; n = 8/8). (**C**) Sarcoplasmic (SR) Ca^2+^ leak quantified as Δ Fura-2 ratio (Iso: 0.021 ± 0.019 Δ Fura-2 ratio vs. Iso + NO_2_-OA: -0.006 ± 0.013 Δ Fura-2 ratio). (**D**) Total SR Ca^2+^ load in Iso versus Iso + NO_2_-OA treated cardiomyocytes (Iso: 0.53 ± 0.29 vs. Iso + NO_2_-OA: 0.56 ± 0.33). (**B–D**) n(cells out of 3 animals) = 8/8. Graphs show Mean ± SEM. Brackets indicate Mean ± SD. ** P < 0.01; *** P < *0.001.*

Analyses of Ca^2+^ levels in isolated cardiomyocytes showed spontaneous Ca^2+^ peaks after RyR2 unblocking upon Iso stimulation (between the red and black cross in Fig. 4A), whereas almost no arrhythmic events could be observed upon additional NO_2_-OA treatment (Fig. 4A, B). Iso treatment resulted in an increased SR-dependent Ca^2+^ leak which was estimated as the difference between the Fura-2 ratio recorded at the end of the 0 Na^+^ /0 Ca^2+^ Tyrode perfusion (black cross) and at the end of the RyR2 inhibitor tetracaine treatment (red cross) (Fig. 4C). Final caffeine treatment revealed that the SR Ca^2+^ load was not significantly elevated after additional NO_2_-OA treatment (Fig. 4D).

Increased SR Ca^2+^ levels have been closely correlated with increased SR Ca^2+^ leak by RyR2-phosphorylation^29,30^. Of interest, caffeine treatment of short-time paced cardiomyocytes (1 Hz) demonstrated equal SR Ca^2+^ loads in vehicle- and NO_2_-OA treated cells. SR Ca^2+^ load was significantly elevated after Iso stimulation, an effect which was slightly, but not significantly, attenuated upon additional NO_2_-OA treatment (Supplemental Figure S3).

### Ca^2+^ sensitivity

**Figure 5:**
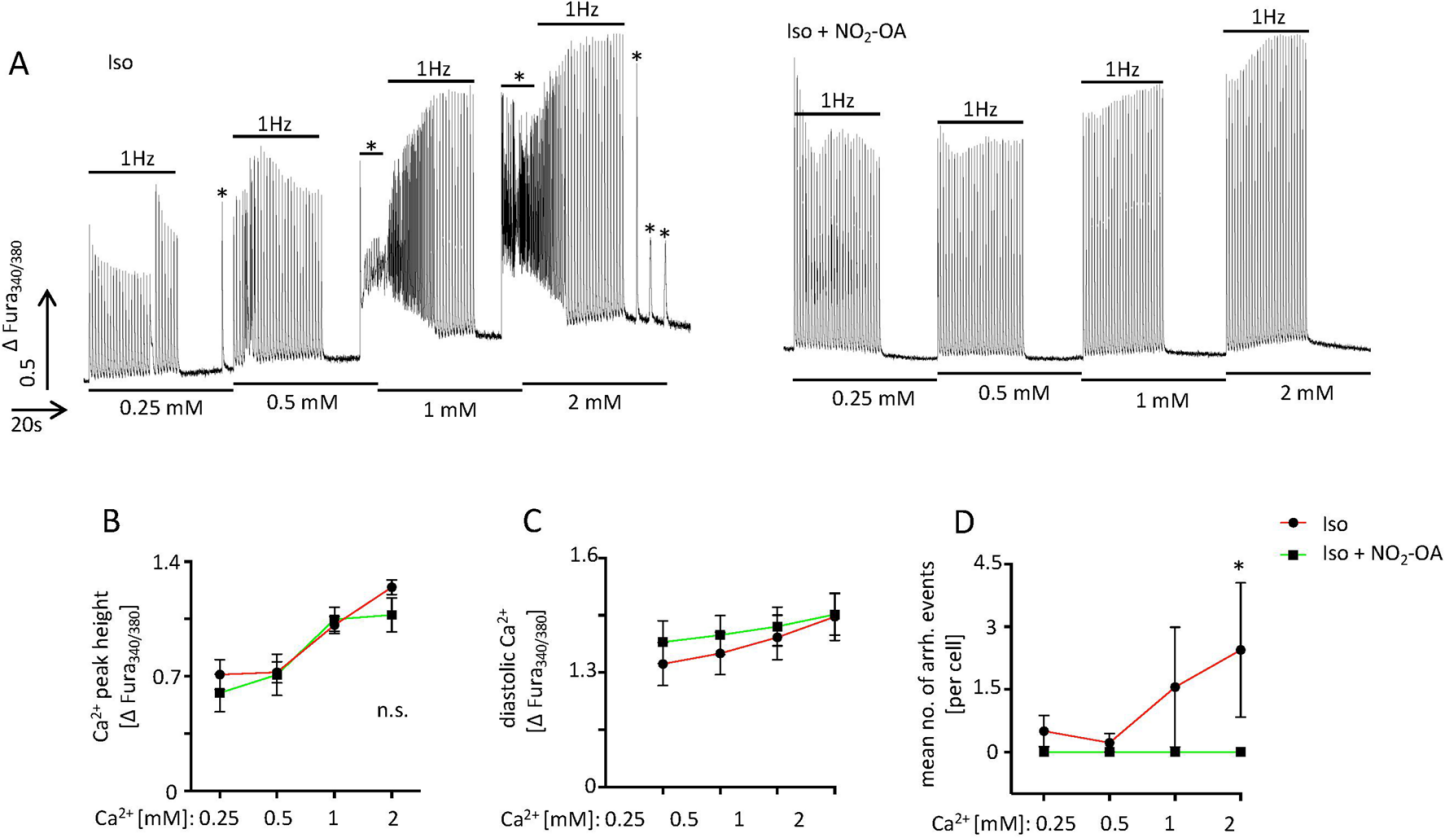
Ca^2+^ sensitivity analyses in isolated adult cardiomyocytes. (**A)** Representative Ca^2+^ transient recordings of isolated adult isoproterenol (Iso)- and Iso + NO_2_-OA treated cardiomyocytes upon increasing extracellular Ca^2+^ concentrations (0.25 mM–2 mM) stimulated at 1 Hz for 30 s following a non-stimulated period of 20 s in which arrhythmic Ca^2+^ events were recorded (asterisks). (**B)** Relative Ca^2+^ transient peak height in control- versus Iso- and Iso + NO_2_-OA treated cells upon different Ca^2+^ concentrations (Iso vs. Iso + NO_2_-OA [∆Fura-2]; 0.25 mM Ca^2+^: 0.71 ± 0.28 vs. 0.6 ± 0.23; 0.5 mM Ca^2+^: 0.73 ± 0.19 vs. 0.71 ± 0.0.28; 1 mM Ca^2+^: 1.01 ± 0.16 vs. 1.05 ± 0.16; 2 mM Ca^2+^: 1.24 ± 0.14 vs. 1.08 ± 0.23). (**C)** Relative diastolic Ca^2+^ levels measured between the 1 Hz pacing periods (Iso vs. Iso + NO_2_-OA [∆Fura-2]; 0.25 mM Ca^2+^: 1.32 ± 0.16 vs. 1.38 ± 0.13; 0.5 mM Ca^2+^: 1.35 ± 0.16 vs. 1.40 ± 0.11; 1 mM Ca^2+^: 1.39 ± 0.16 vs. 1.42 ± 0.11; 2 mM Ca^2+^: 1.45 ± 0.18 vs. 1.45 ± 0.12). (**D)** Mean number of arrhythmic Ca^2+^ release events within the non-stimulated time period (asterisks, Iso vs. Iso + NO_2_-OA [mean number]; 0.25 mM Ca^2+^: 0.5 ± 1.07 vs. 0.0 ± 0.0; 0.5 mM Ca^2+^: 0.22 ± 0.67 vs. 0.0 ± 0.0; 1 mM Ca^2+^: 1.56 ± 4.3 vs. 0.0 ± 0.0; 2 mM Ca^2+^: 2.44 ± 4.82 vs. 0.0 ± 0.0). (**B**–**D**) n(cells out of 3 animals) = 9/5 for each concentration. Graphs show Mean ± SEM. Brackets indicate Mean ± SD. * P < *0.05*.

We further investigated the overall influence of NO_2_-OA on cytosolic Ca^2+^ levels and arrhythmia development upon increasing Ca^2+^ concentrations (representative Ca^2+^ transients are shown in Fig. 5A). Loading of the cells with Ca^2+^ concentrations ranging from 0.25 to 2 mM enhanced systolic calcium transients (Fig. 5B) as well as diastolic Ca^2+^ levels, the latter of which were not affected by additional NO_2_-OA treatment (Fig. 5C). Of importance, NO_2_-OA treatment completely inhibited Iso induced arrhythmic Ca^2+^ release (Fig. 5D).

### Catecholamine-induced CaMKII activity and RyR2 phosphorylation

**Figure 6:**
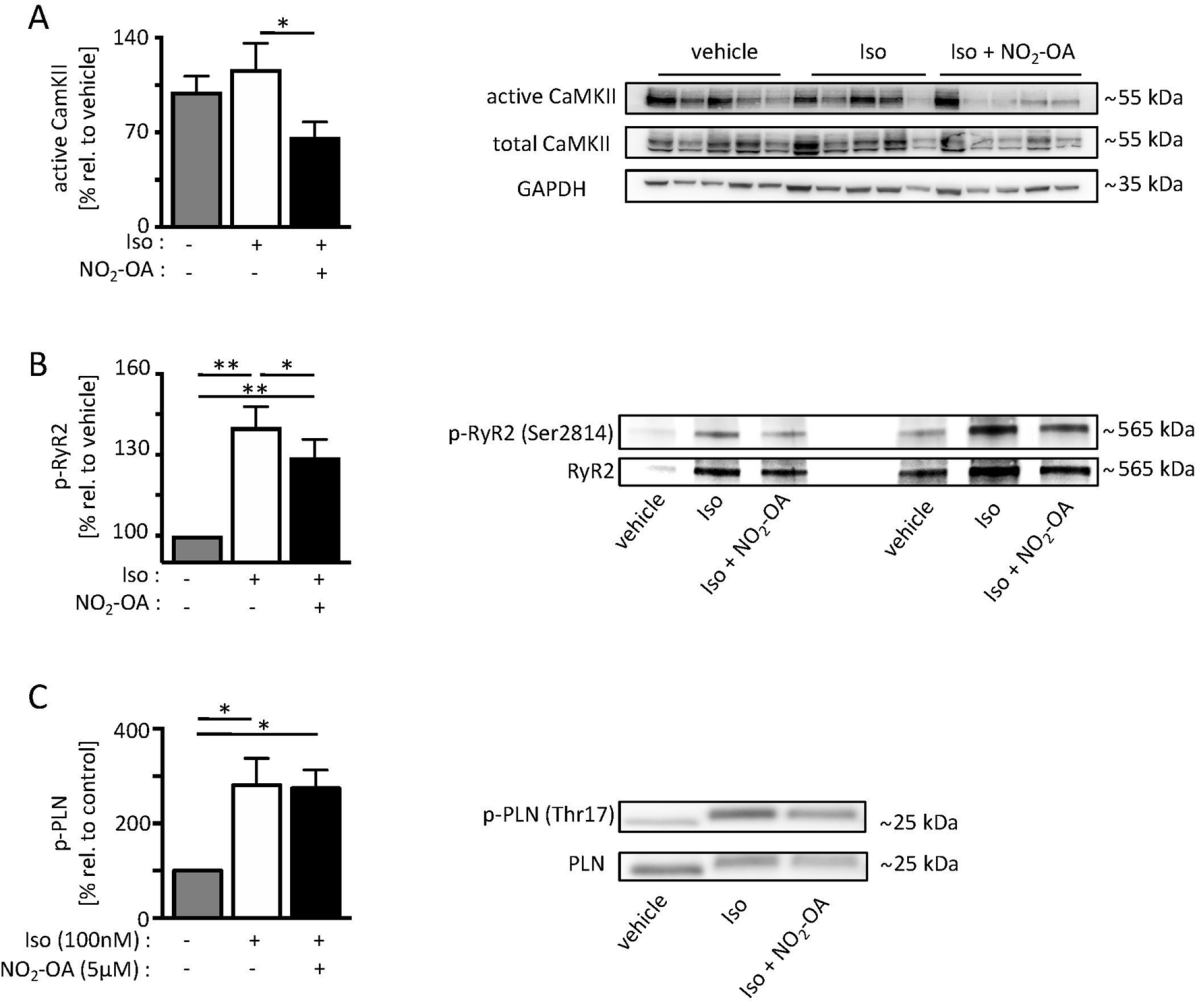
CaMKII activity and RyR2 phosphorylation upon beta-adrenergic stimulation in isolated adult cardiomyocytes. (**A**) Immunoblot analyses after GST pulldown showing active (GST-HDAC4 419-670-bound) CaMKII in relation to total CaMKII (vehicle: 100% ± 36.1 vs. Iso: 116.5% ± 57.9 vs. Iso + NO_2_-OA: 66% ± 34.4). (**B**) Critical RyR2 phosphorylation under Iso stimulation and simultaneous NO_2_-OA treatment as indicated by immunoblotting (vehicle: 100% vs. Iso: 140% ± 22.9 vs. NO_2_-OA: 129% ± 19.8). (**C**) Phosphorylation of phospholamban (Thr17-p-PLN) in vehicle- vs. Iso- vs. Iso + NO_2_-OA treated cardiomyocytes (vehicle: 100% vs. Iso: 107.2% ± 0.1 vs. NO_2_-OA: 107.0% ± 2.9). n (cells out of 3 animals) = 9/9/9. Graphs show Mean ± SEM. Brackets indicate Mean ± SD. * P < 0.05; ** P < 0.01; *** P < *0.001.* The cropped blots are presented, and their full-length blots are included in the Supplemental Figures S4–S6.

Given its importance in RyR2 activity and dysfunction, we next investigated the effect of NO_2_-OA treatment on CaMKII activity by performing a substrate binding assay using GST-HDAC4 419-670, which contains a CaMKII activity-dependent binding site^31^. CaMKII activity of Iso stimulated isolated cardiomyocytes was significantly reduced after NO_2_-OA treatment (Fig. 6A). In accordance, NO_2_-OA markedly diminished the Iso induced increase of pro-arrhythmic RyR2 phosphorylation on Ser2814^32^ as revealed by immunoblotting (Fig. 6B). CaMKII-mediated Thr17-phosphorylation of PLN, which enhances sarcoplasmic reticulum (SR) Ca^2+^-ATPase (SERCA2a) activity^33^, was increased upon Iso treatment and not further affected by NO_2_-OA treatment (Fig. 6C).

## Discussion

Herein, we show that the electrophilic fatty acid nitroalkene NO_2_-OA prevents induction of VT after AMI in vivo by attenuating CaMKII dependent RyR2 leak. These results are of significant clinical relevance since sudden cardiac death related to AMI is most frequently attributed to the occurrence of sustained VT or ventricular fibrillation (VF)^34^. Although the absolute rates of VT and VF have declined during the era of percutaneous coronary intervention, patients suffering from VT or VF prior to revascularization still have a significantly impaired outcome and are at a higher risk for stent thrombosis. These data may underestimate the true incidence, since prehospital SCD before fibrinolysis or any other interventions were not included in this analysis^35^. In the present study, we modeled a clinical scenario of myocardial ischemia by LAD ligation and demonstrated that intraperitoneal injection of NO_2_-OA 20 min before he ischemic episode sustainably reduced ventricular arrhythmic events specifically in the acute phase after AMI.

The prospect of using NO_2_-OA as a therapeutic option for the treatment of acute VT after AMI is underlined by further beneficial effects of this fatty acid nitroalkenes in various cardiovascular diseases. As we and others have demonstrated, the application of NO_2_-OA preserves left ventricular ejection fraction and reduces infarct size in a mouse model of AMI^19,36^. Moreover, NO_2_-OA also protects from angiotensin II induced atrial fibrillation^12^.

Mechanistically, the occurrence of ventricular arrhythmic events during the acute phase of AMI is attributed to development of proarrhythmic substrates with subsequent formation of reentry circuits^3^. The concomitantly enhanced sympathetic tone leads to cellular Ca^2+^ overload, resulting in a disturbed Ca^2+^ homeostasis, accompanied by diastolic Ca^2+^ leak further increasing the vulnerability for VT^37^. On a cellular level, alterations in Ca^2+^ transients have been linked to the emergence of alternans and afterdepolarizations which may trigger VTs^4^, as well as to reduced sodium channel availability resulting in ventricular conduction slowing. This increases the wavelength of reentry circuits thereby increasing the vulnerability to VTs^38–40^.

To investigate the effects of NO_2_-OA on cellular Ca^2+^ transients and sarcoplasmic Ca^2+^ load, both important in arrhythmia development in AMI, we performed analyses of electrically stimulated isolated cardiomyocytes. We did not detect major changes in both Ca^2+^ transient characteristics and in SR Ca^2+^ load upon NO_2_-OA treatment.

We quantified PLN phosphorylation, a protein regulating cytosolic Ca^2+^ clearance into the SR by SERCA2a^5^, at its CaMKII specific phosphorylation site Thr17 under Iso stimulation. Iso increased PLN phosphorylation but no further change was detectable upon treatment with NO_2_-OA. This indicates that NO_2_-OA does not influence cytosolic Ca^2+^ clearance by CaMKII mediated phosphorylation of PLN on this specific site, although we could not fully exclude potential other target sites or phosphorylation mechanisms being modified by NO_2_-OA.

To further examine the underlying molecular effects of NO_2_-OA-mediated VT prevention in detail, we measured RyR2-dependent Ca^2+^ leak from the SR in isolated adult cardiomyocytes treated with Iso. We observed an increased number of arrhythmic events (e.g. EADs and DADs) upon Iso treatment that was markedly reduced by NO_2_-OA. Therefore, we hypothesized that NO_2_-OA beneficially influences CaMKII-dependent modulation of Ca^2+^-homeostasis. Indeed, CaMKII activity and subsequent RyR2 phosphorylation on the critical serine residue (Ser2814), which has been linked to CaMKII-mediated Ca^2+^ leak in cardiomyopathy and catecholamine treatment^41^, was significantly attenuated by NO_2_-OA in Iso-stressed cardiomyocytes (Fig. 6B). These results are in accordance with recent data demonstrating that increased RyR2 phosphorylation and thereby altered Ca^2+^ sensitivity may lead to local Ca^2+^ waves, subsequently depolarizing the membrane potential and forming DADs. These local events furthermore trigger Ca^2+^ waves in adjacent cells and create propagating arrhythmic events^42^.

Speculating on underlying biochemical mechanisms, it is noted that CaMKII activity and subsequent RyR2 phosphorylation is regulated by S-nitrosylation of specific cysteines (e.g. Cys273/ Cys290)^43^. Previous work shows that NO_2_-OA could act as a NO-donor for S-nitrosylation via a modified Nef reaction. This minor and controversial reaction, as opposed to the signaling responses induced by the post-translational alkylation of protein thiols, occurs in very low yields and only yields NO in aqueous buffered solutions when no protein is present^44^. Thus, it is anticipated that under pathological conditions, NO_2_-OA acts via a reversible nitroalkylation of functionally-significant cysteines^11,19^. The further investigation of critical protein targets in this model of cardioprotection will be the subject of further studies.

As this study was performed in a small animal model, a number of limitations may apply when trying to translate the present findings to human pathology. The mechanisms underlying VT in the setting of AMI are the result of multiple alterations, e.g. loss of viable myocardium with loss of cell-to-cell contact, changes in cell membrane potential, ion composition, and other factors predisposing for sudden cardiac death which are only partly mirrored by our experimental setting^3^.

Excessive catecholamine stimulation may be just one of multiple pathways leading to VT development during AMI modulated by NO_2_-OA. However, if other mechanisms, e.g., those responsive to acidosis and cell hypoxia, are altered by NO_2_-OA remains to be investigated.

In summary, we show for the first time that, by inhibiting CaMKII, NO_2_-OA modulates a critical pathway of electrical remodeling following AMI and thus has potential as a pre-emptive anti-arrhythmic strategy for patients at risk for developing AMI.

## Conclusions and perspectives

**Figure 7:**
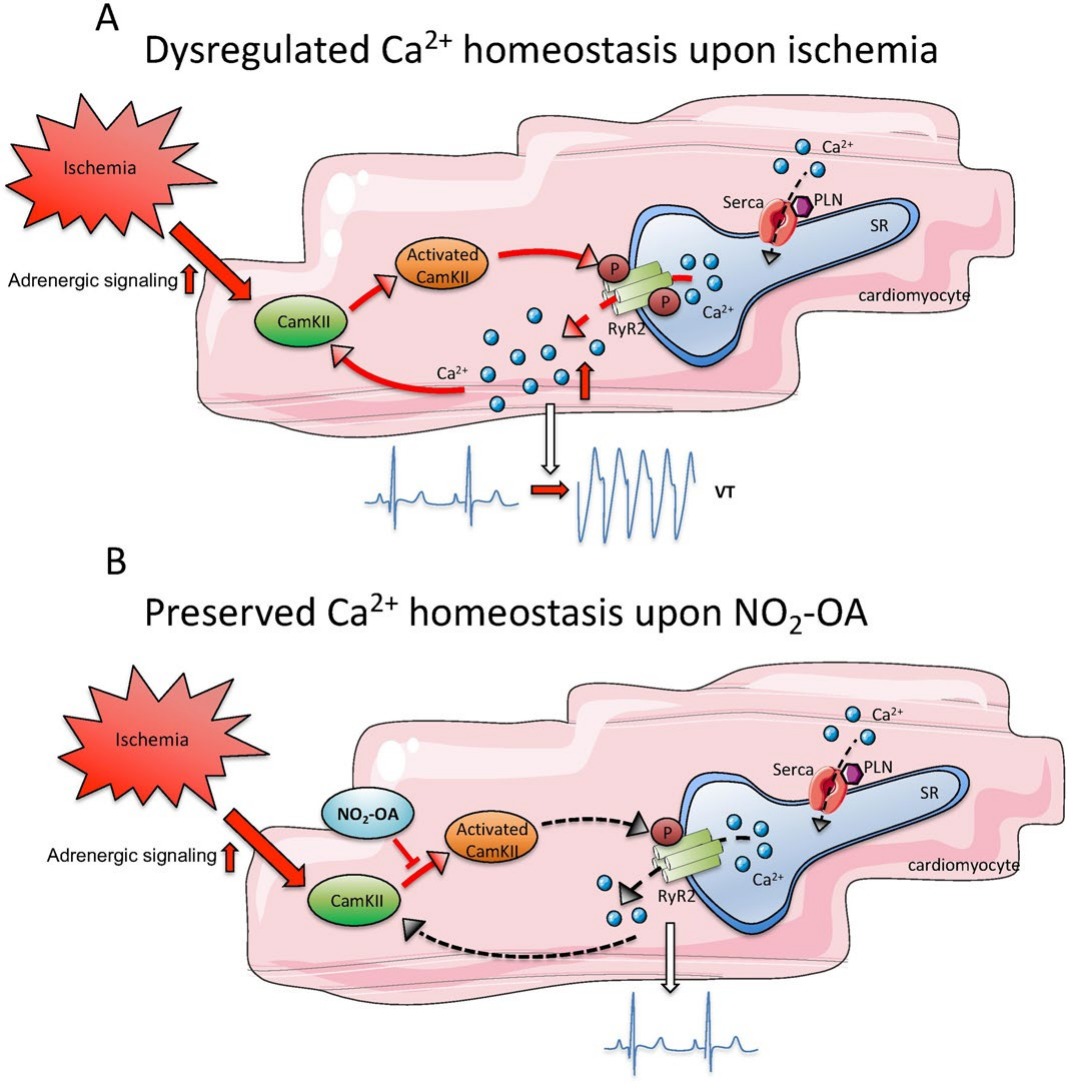
NO_2_-OA prevents acute ischemia induced ventricular tachycardias. (**A**) Increased adrenergic signaling leads to the activation of CaMKII with subsequently dysregulated RyR2-function and impaired Ca^2+^ homeostasis due to a diastolic Ca^2+^ leak. (**B**) Treatment with NO_2_-OA restores Ca^2+^ homeostasis and prevents ventricular tachycardia (VT) development. Figures were produced using Servier Medical Art (https://www.servier.com/).

Our current data reveal that NO_2_-OA rapidly stabilizes myocellular Ca^2+^ homeostasis by inhibition of CaMKII activity in the setting of AMI (Fig. 7). Crucially, herein we show for the first time that, by modulation of this pathway, NO_2_-OA markedly suppresses the susceptibility to ventricular arrhythmias.

There are limited treatment options for prevention of acute arrhythmic tachycardias within the first 72 h after AMI, especially considering the amount of time to reach therapeutic levels for available drugs^45^. In this regard, the long-term dietary supplementation of NO_2_-OA or its precursors, might be a therapeutic option to protect AMI patients against subsequent VT / SCD.

## Materials and methods

An extended description of the methods can be found within the Supplemental Material.

### Animal studies

Male, 8- to 12-week old FVB/N mice (Charles River) were used for all animal studies. All animal studies were approved by the local Animal Care and Use Committees (Ministry for Environment, Agriculture, Conservation and Consumer Protection of the State of North Rhine-Westphalia: State Agency for Nature, Environment and Consumer Protection (LANUV), NRW, Germany) and follow ARRIVE (Animal Research: Reporting of In Vivo Experiments) guidelines.

### Left anterior descending artery (LAD) ligation

LAD ligation to induce myocardial ischemia was performed as described by Mollenhauer et al.^26^.

### Right ventricular stimulation

A detailed stimulation protocol can be found within the Supplemental Material. In short, right ventricular stimulation was performed 20 min after induction of myocardial ischemia, while the mouse was kept under anesthesia, according to a standardized protocol that initially included a programmed ventricular stimulation with a fixed S1S1 interval to whose last S1-impulse a short-coupled additional stimulus was applied. After 10 s of recovery, automated burst stimulation was performed according to the protocol. VT were defined as a series of repetitive ventricular ectopic beats lasting for > 200 ms^26^.

### In vivo electrophysiological mapping

In brief, directly after electrophysiological investigation the heart was exposed by thoracotomy. A 32-electrode microelectrode array (MEA, Multichannel Systems, Reutlingen, Germany) was positioned on the epicardial surface of the left ventricle apico-septally of the peri-ischemic region as described previously^26^. Field potentials were recorded using a 128-channel, computer-assisted recording system (Multichannel Systems). Data was evaluated according to Lammers et al.^46^. In short, inter-electrode conduction latencies (reciprocal conduction velocity) were calculated for all neighboring electrodes. Of each neighboring quadruplet of electrodes, the largest latency was taken and plotted as a phase map. From this phase map the mean latency of conduction was calculated. The variation coefficient of the phase map was calculated (Percentile (P): P_5–95_/P_50_) to receive the index of inhomogeneity as velocity independent factor of conduction inhomogeneity. Phase maps were calculated using custom-programmed software (Excel).

### Administration of nitro-oleic acid

Nitro-oleic acid (9- and 10-nitro-octadec-9-cis-enoic acid; NO_2_-OA), administered at 20 nmol/g body weight, was solvated in polyethylene-glycol/ethanol (85:15, vol/vol), or 100 ml of vehicle (polyethylene-glycol/ ethanol, 85:15). These preparations were injected i.p. 20 min prior to LAD ligation. NO_2_-OA was synthesized as previously described^47^.

### Isolation of adult ventricular cardiomyocytes

Ventricular myocytes were obtained from 8 to 12 week old male wild-type mice (FVB/N) as described previously^28^.

### Assessment of Ca^2+^ transients

All experiments were performed at 37 °C within 6 h after cell isolation, as described previously using ratiometric Ca^2+^ imaging with Fura-2 dye (IonOptix)^28^.

For Ca^*2*+^ *transient analyses* the cells were incubated with either 0.1% EtOH as control, 10 µM Iso in 0.1% EtOH or 10 µM Iso and 5 µM NO_2_-OA in 0.1% EtOH for 10 min. Calcium transients were recorded under electrically stimulated biphasic field pulses (20 V, 4 ms) at a frequency of 1 Hz for 1 min.

*SR Ca*^*2*+^ *leak and load* were measured according to a modified protocol^28,48^. Fura-2 loaded ventricular cardiomyocytes were incubated with 10 µM Iso for 7 min and stimulated for 3 min at 1 Hz, 20 V, 4 ms until cellular Ca^2+^ transients reached a steady state followed by a burst stimulation with 4 Hz for 30 s. Directly after the last pulse the pacing was stopped and the normal Tyrode solution was substituted by a 0 Na^+^ /0 Ca^2+^ Tyrode supplemented with 10 mmol/l EGTA and 1 mmol/l of the RyR2 inhibitor tetracaine in which Na^+^ was replaced by Li^+^. This condition allowed measuring intracellular Ca^2+^ levels in a closed system without trans-sarcolemmal Ca^2+^ fluxes and prevents SR Ca^2+^ leak into the cytoplasm. After 40 s recording time the solution was switched back to 0 Na^+^ /0 Ca^2+^ Tyrode without tetracaine for another 40 s to unblock the ryanodine receptors and allow potential Ca^2+^ leak. SR Ca^2+^ leak was estimated as the difference between the Fura-2 ratio recorded at the end of the 0 Na^+^ /0 Ca^2+^ Tyrode perfusion with and without tetracaine. At the end of the protocol, 10 mM caffeine was applied to evaluate the total SR Ca^2+^ content.

*Ca*^*2*+^ *sensitivity:* Fura-2 loaded ventricular cardiomyocytes were incubated with Tyrode solution containing 0.25 mM, 0.5 mM, 1 mM or 2 mM of Ca^2+^ together with either 10 µM Iso in 0.1% EtOH or 10 µM Iso and 5 µM NO_2_-OA in 0.1% EtOH. For each calcium concentration cells were paced at 1 Hz for 30 s following a 20 s non-pacing period in which pro-arrhythmic calcium release events was counted. Ca^2+^ transient height was determined within the last paced Ca^2+^ transient before the non-paced period.

Ca^2+^ transient characteristics were calculated by the IonWizard (IonOptix) software.

### Immunoblotting

Protein extraction and immunoblotting were performed as previously described^26^. Briefly, membranes were incubated with the following antibodies overnight at 4 °C: anti-GAPDH (1:1000; Santa Cruz), anti-P-PLN-Thr17, anti-PLN, anti-P-RyR2-Ser2814 (1:5000; Badrilla), anti-RyR2 (1:2000; Sigma-Aldrich). Full blots are shown in the Supplemental Material section.

### CaMKII activity assay

CaMKII activity was assessed by detecting the amount of endogenous CaMKII that binds to GST-HDAC4 amino acids 419 to 670 (containing the CaMKII activity–dependent binding domain) as described previously^31^. Bound active CaMKII, CaMKII input, and GST-HDAC4 419-670 input were resolved by SDS-PAGE and detected by immunoblot using HDAC4 (1:1000; Santa Cruz) and CaMKII antibodies (1:1000; BD Bioscience). Protein loading was normalized to GAPDH (1:10 000; Sigma-Aldrich).

### Statistical analysis

Results are expressed as mean ± standard error of the mean. Gaussian normality was tested via Shapiro–Wilk normality test. Unpaired Student’s t-test or one-way ANOVA were used for gaussian distributed data, whereas non-gaussian distributed data were analyzed using Kruskal Wallis test, each followed by appropriate post-hoc test. Results of Fig. 5B, C are normalized against their respective control within the same animal. An alpha level of *P* < 0.05 was considered statistically significant. All statistical calculations were carried out using GraphPad Prism 8.2.1 (GraphPad). *P < 0.05, **P < 0.01, ***P < 0.001.

## Supplementary information

Supplementary information.
