# Supplementary material for: Nitro-fatty acids suppress ischemic ventricular arrhythmias by preserving calcium homeostasis
Source: Sci Rep. 2020 Sep 18;10:15319. doi: 10.1038/s41598-020-71870-6 (PMC7501300; doi:10.1038/s41598-020-71870-6)
Supplement: Supplementary file 1 — Supplementary information. [file 41598_2020_71870_MOESM1_ESM.pdf]

Nitro-fatty acids suppress ischemic ventricular arrhythmias by preserving calcium homeostasis

Martin Mollenhauer*[1], Dennis Mehrkens*[1], Anna Klinke[2], Max Lange[1], Lisa Remane[1], Kai Friedrichs[2], Simon Braumann[1], Simon Geißen[1], Sakine Simsekyilmaz[1], Felix Nettersheim[1], Samuel Lee[1], Gabriel Peinkofer[1], Anne C. Geisler[3], Bianca Geis[3], Alexander Peter Schwoerer[4], Lucie Carrier[5], Bruce A. Freeman[6], Matthias Dewenter[7], Xiaojing Luo[8], Ali El-Armouche[8], Michael Wagner[8], Matti Adam[1], Stephan Baldus[1], Volker Rudolph[2]

[1] Clinic III for Internal Medicine, Department of Cardiology, Center for Molecular Medicine Cologne (CMMC) University of Cologne, Cologne, Germany and Center for Molecular Medicine Cologne, CMMC, University of Cologne, Cologne, Germany
[2] Clinic for General and Interventional Cardiology/ Angiology, Herz- und Diabeteszentrum NRW, Ruhr-Universitaet Bochum, Bad Oeynhausen, Germany
[3] General and Interventional Cardiology University Heart Center Hamburg, University Hospital Hamburg-Eppendorf (UKE), Germany
[4] Department of Cellular and Integrative Physiology, University Medical Center Hamburg Eppendorf, DZHK (German Centre of Cardiovascular Research), partner site Hamburg/Kiel/Lübeck, Hamburg, Germany
[5] Experimental Pharmacology and Toxicology, University Hospital Hamburg-Eppendorf (UKE), Hamburg, Germany
[6] Department of Pharmacology and Chemical Biology, University of Pittsburgh, Pittsburgh, PA, USA
[7] Institute of Experimental Cardiology, University of Heidelberg, Heidelberg, Germany; and German Centre for Cardiovascular Research (DZHK), Partner Site, Heidelberg/Mannheim, Germany
[8] Department of Pharmacology and Toxicology, Technische Universitaet Dresden, Dresden, Germany

*both authors contributed equally

Corresponding author: Dr. rer. nat. Martin Mollenhauer
Kerpener Str. 62
50937 Köln, Germany
Tel: +49-221-478-87402
Fax: +49-221-478-8737 2
E-Mail: martin.mollenhauer@uk-koeln.de

Supplemental Materials

Administration of nitro-oleic acid

Nitro-oleic acid (NO_2-OA) was provided from the lab of Bruce Freeman, PhD, University of Pittsburgh. 20 nmol/g bodyweight NO_2-OA in polyethylen-glycol/ethanol (85:15, vol/vol) or 100 ml of vehicle (polyethylenglycol/ ethanol, 85:15) were injected 20 min i.p. prior to ligation of the left anterior descending artery (LAD). NO_2-OA was synthesized as previously described[1].

Right ventricular stimulation

Right ventricular stimulation was performed 20 minutes after induction of ischemia (ligation of the LAD) while mice were kept under anaesthesia and placed in supine position on a heating pad. An octapolar electrophysiological catheter (1.1 F, Scisense) was inserted via the right jugular vein to the right atrium and ventricle. Surface ECG was analyzed under stable baseline conditions for at least 3 min. Electrophysiological investigation with induction of arrhythmias was performed as described previously[2]. Programmed ventricular stimulation was performed at a pacing stimulus amplitude of 1.0 mA with 7 stimuli fixed rate at S1S1 cycle length of 120 ms, 110 ms and 100 ms, respectively, with one short coupled extra stimulus with a 10 ms-stepwise S1S2 reduction starting at cycle length of 80 ms down to 10 ms (24 stimuli in total, see below).

Stepwise reduction protocol S1S1/S1S2		
1. 120/80	9. 110/80	17. 100/80
2. 120/70	10. 110/70	18. 100/70
3. 120/60	11. 110/60	19. 100/60
4. 120/50	12. 110/50	20. 100/50
5. 120/40	13. 110/40	21. 100/40
6. 120/30	14. 110/30	22. 100/30
7. 120/20	15. 110/20	23. 100/20
8. 120/10	16. 110/10	24. 100/10

After 10 sec recovery period, ventricular burst stimulation was performed for 1 sec (three times consecutively) at S1S1 stimulation cycle lengths starting at 50 ms with stepwise

reduction down to 10 ms at pacing stimulus amplitudes of 1.0 mA (15 stimuli in total, see below).

Burst stimulation - protocol S1S1
1. 20*50
2. 20*50
3. 20*50
4. 25*40
5. 25*40
6. 25*40
7. 33*30
8. 33*30
9. 33*30
10. 50*20
11. 50*20
12. 50*20
13. 100*10
14. 100*10
15. 100*10

Ventricular tachycardia (VT) was defined as a series of repetitive ventricular ectopic beats lasting for >200 ms.

In vivo electrophysiological mapping

Directly after electrophysiological investigation mice were kept anaesthetized with isoflurane and placed in supine position on a heating pad. The heart was exposed by thoracotomy. A 32-electrode microelectrode array (MEA, Multichannel Systems, Reutlingen, Germany) was positioned on the epicardial surface of the left ventricle apico-septally of the periischemic region as described previously[2]. Field potentials were recorded using a 128-channel, computer-assisted recording system (Multichannel Systems) with a sampling rate of 25 kHz. Data were bandpass filtered (50 Hz), digitized with 12 bit and a signal range of 20 mV. The first derivative of each unipolar field potential was evaluated and maximal slope of dV/dt activation was defined as timepoint of local activation for spontaneous stimulation[3]. The

activation time differences to neighbouring points were normalized to inter-electrode distance (300 µm for vertical and horizontal adjacent electrodes) to receive the conduction velocity.

Data was further evaluated according to Lammers et al.[4]: Inter-electrode conduction latencies (reciprocal conduction velocity) were calculated for all neighboring electrodes. Of each neighboring quadruplet of electrodes the largest latency was taken and plotted as a phase map. From this phase map the mean latency of conduction was calculated. The variation coefficient of the phase map was calculated (Percentile (P): P_{5-95} / P_{50}) and used as index of inhomogeneity to receive a velocity independent factor of conduction inhomogeneity. Phase maps were calculated using custom-programmed software (Excel)[4–6].

Isolation of adult ventricular cardiomyocytes

Ventricular myocytes were obtained from 8 to 12-week old male mice (FVBN). Animals were anesthetized by intraperitoneal injection of pentothal (150 mg/kg), and the heart was quickly removed and placed into cold Ca^{2+}-free Tyrode's solution containing (in mmol/l): NaCl 113, KCl 4.7, $MgSO_4$ 4, KH_2PO_4 0.6, NaH_2PO_4 0.6, BDM 10, $NaHCO_3$ 1.6, HEPES 10, Taurine 30, D-glucose 20, adjusted to pH 7.4, 37 °C. The ascending aorta was cannulated, and the heart was perfused with oxygenated Ca^{2+}-free Tyrode's solution at 37°C during 4 min. For enzymatic dissociation, the heart was perfused with Ca^{2+}-free Tyrode's solution containing liberase TM research grade (Roche Diagnostics) for 10 min at 37°C. Then the heart was removed and placed into a dish containing Tyrode's solution supplemented with 0.2 mmol/l $CaCl_2$ and 5 mg/ml BSA (Sigma-Aldrich). The ventricles were separated from the atria, cut into small pieces, and triturated with a pipette to disperse the myocytes. Ventricular myocytes were filtered on gauze and allowed to sediment by gravity for 10 min. The supernatant was removed and cells were suspended in Tyrode's solution supplemented with 0.5 mmol/l $CaCl_2$ and 5 mg/ml BSA. The procedure was repeated once and cells were suspended in Tyrode's solution with 1 mmol/l $CaCl_2$. Freshly isolated ventricular myocytes were plated in 35 mm culture dishes

coated with laminin (10 µg/ml) and stored at 37 °C until use. The protocol was adapted from Vettel et al.[7].

Patch-clamp experiments

Investigated cells were rod-shaped, had a clearly visible cross striation and showed no spontaneous contractions. Experiments were performed at 37°C and cells were kept in Tyrode solution (mmol/l: 2 $CaCl_2$, 10 Glucose, 10 HEPES, 4 KCl, 1 $MgCl_2$, 138 NaCl, 0.33 NaH_2PO_4, pH 7.3). The patch-pipette contained a potassium-glutamate solution (mmol/l: 1.1 ATP, 10 EGTA, 120 glutamic acid, 10 HEPES, 10 KCl, 2 $MgCl_2$, pH7.2). Whole-cell patch-clamp measurements were performed using ruptured patch technique and an EPC 9 patch-clamp amplifier (HEKA Elektronik). The cells were stimulated briefly with 5 ms or 10 ms depolarization currents respectively, with 150 % specific AP threshold and a frequency of 1 Hz at baseline. For the final experiments, cells were stimulated for 1 minute in Tyrode solution followed by stimulation for 5 minutes in Tyrode supplemented with 0.1% EtOH (control) followed by 5 minutes stimulation in 5 µmol/l NO_2-OA / 0.1 % EtOH. Data were low pass filtered at 1 kHz and analyzed using the FitMaster software (version 2.x60, HEKA Elektronik).

Assessment of Calcium transients

All experiments were performed at 37°C within 6 hrs after cell isolation. Cells were washed with Tyrode ((in mmol/l): KCl 5.4; NaCl 121.6; Na-pyruvate 5; $NaHCO_3$ 4.013; NaH_2PO_4 0.8; $CaCl_2$ 1.0; $MgCl_2$ 1.8; glucose 5 and HEPES 10 (pH 7.4 with NaOH)) and transferred to Tyrode solution containing 5 µM Fura-2-AM for 20 min, washed again with Tyrode solution let them recover for 10 min at 37°C. Cells for analyses were selected according to the following criteria: rod-shaped, no membrane blebs, no hypercontractile zones, no spontaneous contractions, and stable contraction amplitude at 1 Hz. Sarcomere shortening and Fura-2 ratio (measured at 512 nm upon excitation at 340 and 380 nm) were simultaneously recorded by using a double excitation pectrofluorimeter coupled with a video detection system (IonOptix, Milton, MA, USA). The F340/F380 ratio was used as an index of cytosolic Ca^{2+}

concentration. For calcium transient analyses and simultaneous assessment of sarcomere shortening the cells were incubated with either 0.1% EtOH, 10 μM Iso in 0.1% EtOH or 10 μM Iso and 5μM NO_2-OA in 0.1% EtOH for 10 minutes. Calcium transients and sarcomere shortening was recorded under electrically stimulated biphasic field pulses (20 V, 4 ms) at a frequency of 1Hz for 1min.

SR Ca^{2+} load: Fura-2 loaded ventricular cardiomyocytes were incubated either with 0.1% EtOH as vehicle, 5 μM NO_2-OA in 0.1% EtOH, 10 μM Iso in 0.1% EtOH or 10 μM Iso and 5 μM NO_2-OA in 0.1% EtOH for 10 minutes and stimulated at 1Hz for 20 seconds. Pacing was stopped for 10 seconds and 10 mM caffeine was applied to evaluate the total SR Ca^{2+} content.

Assessment of Calcium leak

SR Ca^{2+} leak and load were measured according to a modified protocol.[7,8] Fura-2 loaded ventricular cardiomyocytes were incubated with 10 μM Iso for 7 min and stimulated for 3 min at 1 Hz, 20 V, 4 ms until cellular Ca^{2+} transients reached a steady state followed by a burst stimulation with 4 Hz for 30 sec. Directly after the last pulse the pacing was stopped and the normal Tyrode solution was substituted by a 0 Na^{+} /0 Ca^{2+} Tyrode supplemented with 10 mmol/l EGTA and 1 mmol/l of the RyR2 inhibitor tetracaine in which Na^{+} was replaced by Li^{+}. This condition allowed measuring intracellular Ca^{2+} levels in a closed system without trans-sarcolemmal Ca^{2+} fluxes and prevents SR Ca^{2+} leak into the cytoplasm. After 40 sec recording time the solution was switched back to 0 Na^{+} /0 Ca^{2+} Tyrode without tetracaine for another 40 sec to unblock RyR2 and allow a potential Ca^{2+} leak. SR Ca^{2+} leak was estimated as the difference between the Fura-2 ratio recorded at the end of the 0 Na^{+} /0 Ca^{2+} Tyrode perfusion with and without tetracaine. At the end of the protocol, 10 mM caffeine was applied to evaluate the total SR Ca^{2+} content.

CaMKII activity assay

CaMKII activity was assessed by detecting the amount of CaMKII that binds to GST-HDAC4 amino acids 419 to 670 (containing the CaMKII activity–dependent binding domain) as described previously.[9] Bound active CaMKII, CaMKII input, and HDAC4 input were resolved by SDS-PAGE and detected by Western blot using HDAC4 (1:1000; Santa Cruz) and CaMKII antibodies (1:1000; BD Bioscience). Protein loading was normalized to GAPDH (1:10 000; Sigma-Aldrich).

Immunoblotting

Protein extraction and Western blotting was performed as previously described according to standard protocols.[2] Briefly, membranes were blocked with Roti-Block (Carl Roth) for 1 hour and afterwards incubated with the following antibodies overnight at 4°C: anti-GAPDH (1:1000; Santa Cruz), anti-P-PLN-Thr17, anti-PLN, anti-P-RyR2-Ser2814 (1:5000; Badrilla), anti-RyR2 (1:2000; Sigma-Aldrich). After incubation with appropriate secondary antibodies for 1 hour, chemiluminescence was detected using a VersaDoc (Biorad) or Fusion FX (Vilber Lourmat) imaging system and quantified with Quantity One (Biorad). Protein extraction and Western blotting was performed as previously described according to standard protocols.

References

1. Schopfer, F. J. *et al.* Fatty Acid Transduction of Nitric Oxide Signaling. *J. Biol. Chem.* **280**, 19289–19297 (2005).

2. Mollenhauer, M. *et al.* Myeloperoxidase Mediates Postischemic Arrhythmogenic Ventricular Remodeling. *Circ. Res.* CIRCRESAHA.117.310870 (2017). doi:10.1161/CIRCRESAHA.117.310870

3. Coronel, R. *et al.* Laplacian electrograms and the interpretation of complex ventricular activation patterns during ventricular fibrillation. *J. Cardiovasc. Electrophysiol.* **11**, 1119–28 (2000).

4. Lammers, W. J., Schalij, M. J., Kirchhof, C. J. & Allessie, M. A. Quantification of spatial inhomogeneity in conduction and initiation of reentrant atrial arrhythmias. *Am. J. Physiol.* **259**, H1254-63 (1990).

5. Schrickel, J. W. *et al.* Enhanced heterogeneity of myocardial conduction and severe cardiac electrical instability in annexin A7-deficient mice. *Cardiovasc. Res.* **76**, 257–68 (2007).

6. Li, D., Fareh, S., Leung, T. K. & Nattel, S. Promotion of atrial fibrillation by heart failure in dogs: atrial remodeling of a different sort. *Circulation* **100**, 87–95 (1999).

7. Vettel, C. *et al.* Phosphodiesterase 2 Protects Against Catecholamine-Induced Arrhythmia and Preserves Contractile Function After Myocardial Infarction. *Circ. Res.* **120**, 120–132 (2017).

8. Shannon, T. R., Ginsburg, K. S. & Bers, D. M. Quantitative assessment of the SR Ca2+ leak-load relationship. *Circ. Res.* **91**, 594–600 (2002).

9. Dewenter, M. *et al.* Calcium/Calmodulin-Dependent Protein Kinase II Activity Persists During Chronic β-Adrenoceptor Blockade in Experimental and Human Heart Failure. *Circ. Hear. Fail.* **10**, (2017).

Supplemental Figures

S1

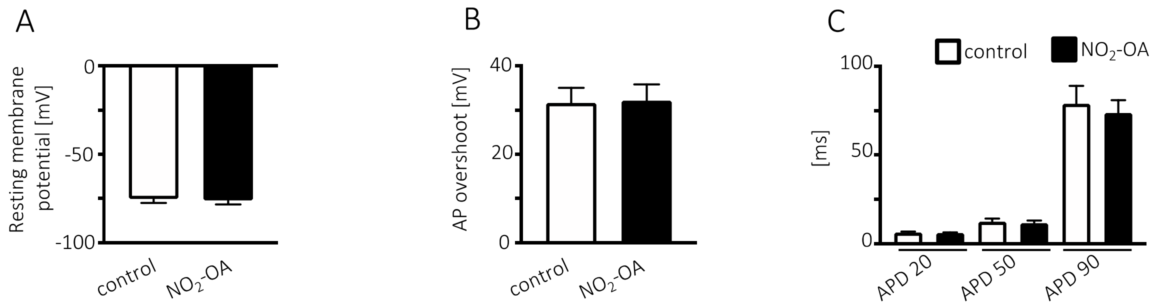

Supplemental Figure 1: Action potential analyses via ruptured-patch whole-cell patch-clamp analyses of isolated adult cardiomyocytes. **A**: Analyses revealed no differences in resting membrane potential (control: -75.33 ± 4.8 vs. NO_2-OA: -76.15 ± 5.0 mV; p=0.8; n=5/5) , **B**: AP overshoot (control: 31.62 ± 7.7 vs NO_2-OA: 32.18 ± 0.04 mV; p=0.91; n=5/5) and **C**: APD 20 (control: 6.31 ± 1.1 vs. NO_2-OA: 5.96 ± 1.0 ms; p>0.99 ; n=5/5), APD 50 (control: 12.44 ± 3.87 vs. NO_2-OA: 11.65 ± 3.13 ms; p>0.99 ; n=5/5; C) and APD 90 (control: 78.76 ± 22.63 vs. NO_2-OA: 73.70 ± 16.08 ms; p=0.98; n=5/5) between control and NO_2-OA treated cells. APD: Action potential duration.

S2

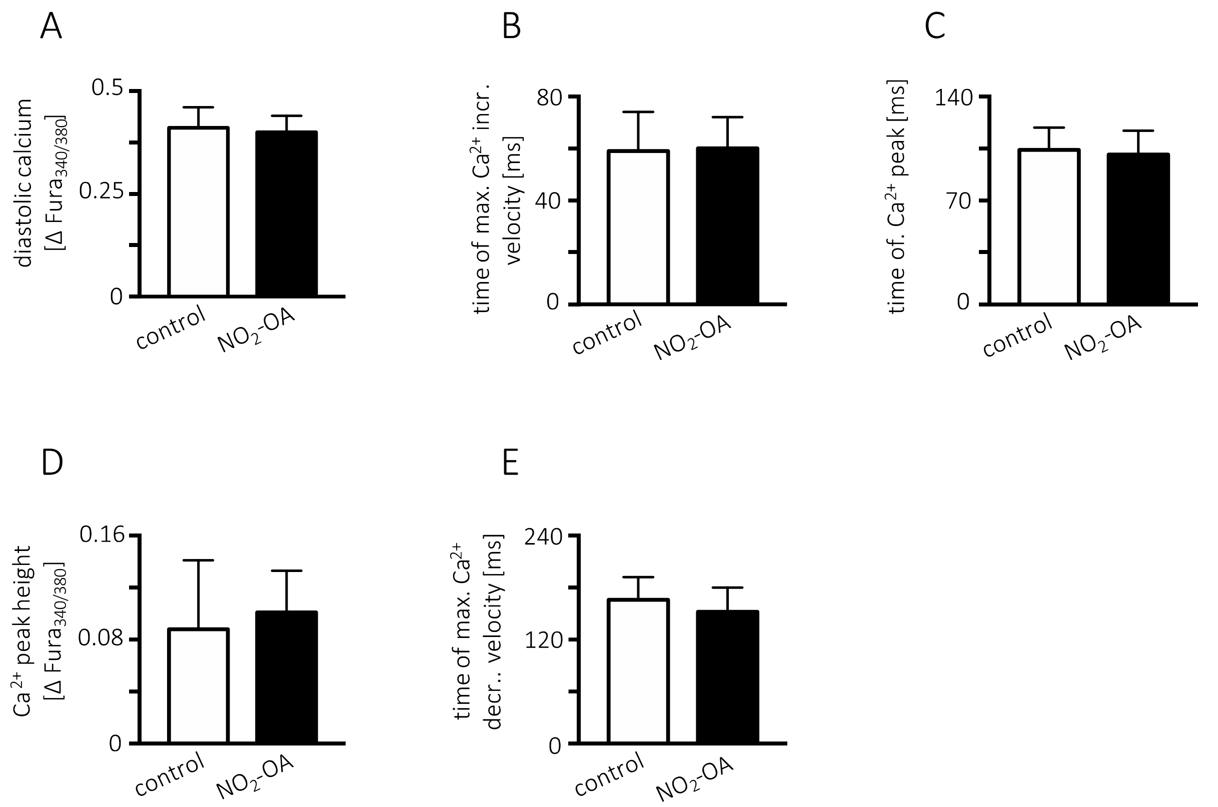

Supplemental Figure 2. Ca^{2+} transients under baseline conditions: No differences in characteristics of Ca^{2+} transient was noted upon treatment with **NO_2-OA*.*** **A**: In detail, no difference in diastolic calcium concentration was noted between groups (control: 0.41 ± 0.05 vs. NO_2-OA: 0.40 ± 0.04 ratio units; p=0.55; n=11/11). **B**: Moreover, time-to-maximum velocity of Ca^{2+} increase (control: 59 ± 15 vs. 60 ± 12 ms; p=0.87; n=11/11), **C**: time to peak Ca^{2+} concentration (control: 104 ± 15 vs. NO_2-OA: 101 ± 16ms; p=0.78; n=11/11), **D**: the peak height of the Ca^{2+} transient (control: 0.088 ± 0.053 vs. NO_2-OA: 0.101 ± 0.032 ratio units; p=0.45; n=11/11) and **E**: the time to maximum velocity of Ca^{2+} decrease (control: 166 ± 26 vs. NO_2-OA: 152 ± 28 ms; p=0.21; n=11/11) did not differ between groups.

S3

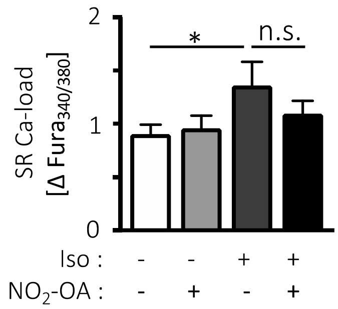

Supplemental Figure 3. Sarcoplasmic Ca^{2+} load analysis in isolated adult cardiomyocytes. Total SR Ca^{2+} load analyses of control- vs. NO_2-OA- vs. Iso vs. Iso + NO_2-OA treated cardiomyocytes after 20 seconds 1Hz pacing followed by 10 seconds without pacing followed by SR Ca^{2+} release by caffeine. Evaluation of the Fura-2 ratio revealed elevated SR Ca^{2+} load after Iso treatment with no significant changes upon additional NO_2-OA treatment. Of note, SR Ca^{2+} load was not changed after NO_2-OA treatment at baseline conditions. vehicle: 0.9 ± 0.21 ΔFura-2 vs. NO_2-OA: 1.05 ± 0.38 ΔFura-2 vs. Iso: 1.36 ± 0.50 ΔFura-2 vs. Iso + NO_2-OA: 1.02 ± 0.37 ΔFura-2; n(cells out of 3 animals) = 6/7/5/5. Graphs show Mean ± SEM. Brackets indicate Mean ± SD. * *$P < 0.05$.*

S4

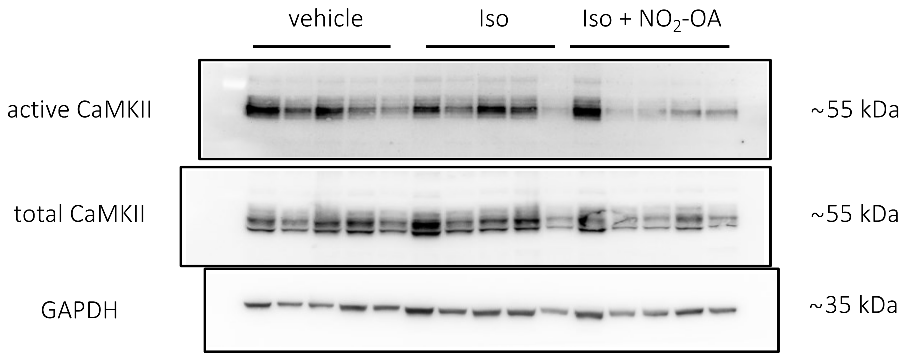

Supplemental Figure 4. Full immunoblots of Figure 6A.

S5

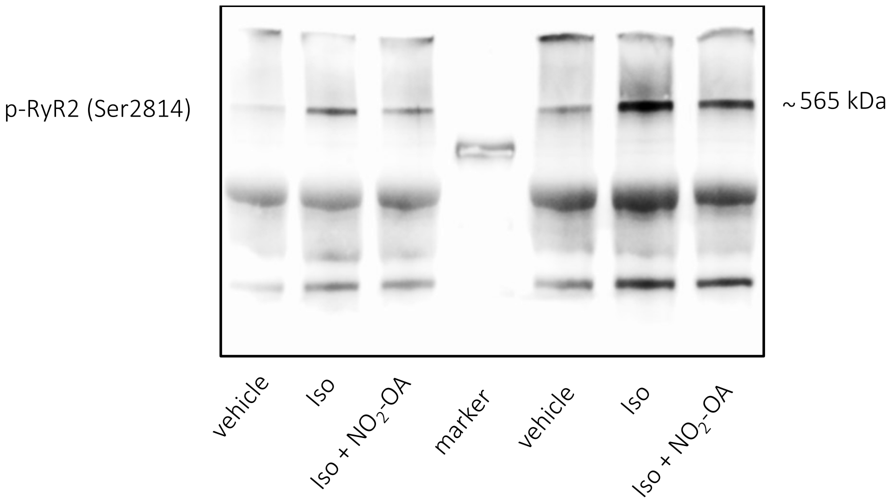

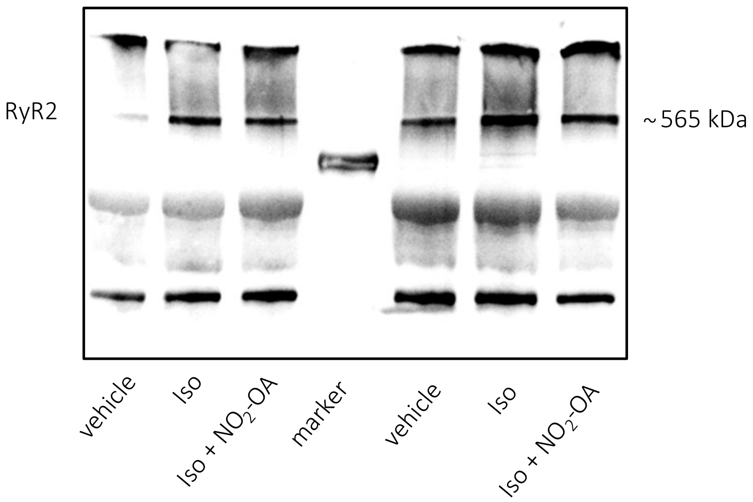

Supplemental Figure 5. Full immunoblots of Figure 6B.

S6

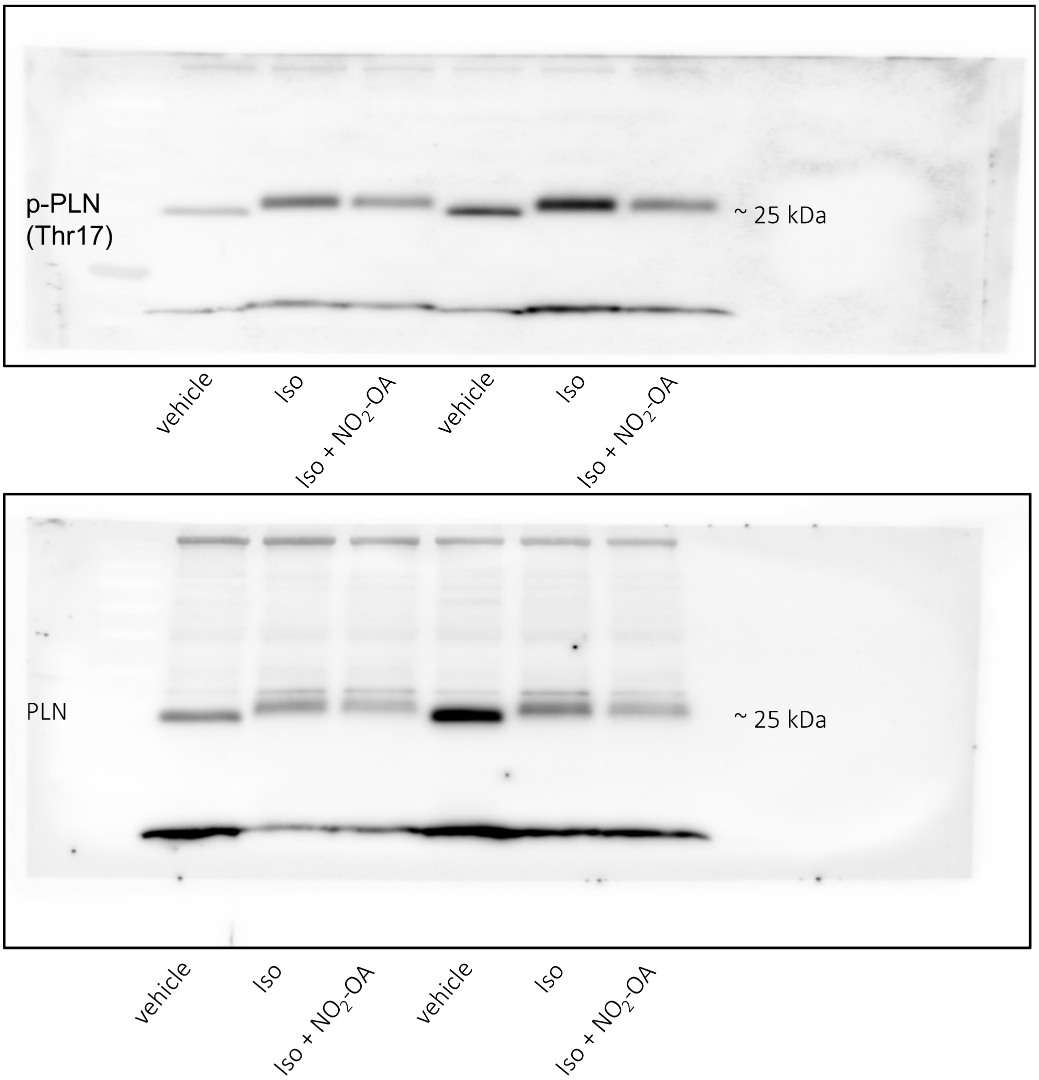

Supplemental Figure 6. Full immunoblots of Figure 6C.
